# Analysis of the Serum Profile of Cytokines Involved in the T-Helper Cell Type 17 Immune Response Pathway in Atopic Children with Food Allergy

**DOI:** 10.3390/ijerph19137877

**Published:** 2022-06-27

**Authors:** Kacper Packi, Joanna Matysiak, Sylwia Klimczak, Eliza Matuszewska, Anna Bręborowicz, Dagmara Pietkiewicz, Jan Matysiak

**Affiliations:** 1 Department of Inorganic and Analytical Chemistry, Poznan University of Medical Sciences, 60-780 Poznan, Poland; kacperpacki1@wp.pl (K.P.); eliza.matuszewska@gmail.com (E.M.); dagmarapietkiewicz3@gmail.com (D.P.); 2 AllerGen, Center of Personalized Medicine, 97-300 Piotrkow Trybunalski, Poland; sylwia.cichuta@gmail.com; 3 Faculty of Health Sciences, Calisia University-Kalisz, 62-800 Kalisz, Poland; jkamatysiak@gmail.com; 4 Department of Nucleic Acid Biochemistry, Medical University of Lodz, 251 Pomorska Str., 92-213 Lodz, Poland; 5 Department of Pulmonology, Pediatric Allergy and Clinical Immunology, Poznan University of Medical Sciences, 60-572 Poznan, Poland; abreborowicz@wp.pl

**Keywords:** IL-1 beta, IgE-mediated food allergy, atopic dermatitis, cytokines

## Abstract

The main risk factor for the development of food allergies (FAs) in children is atopic dermatitis (AD). AD is usually recognized as the Th1/Th2 paradigm of allergic disease. Recently, the Th1/Th2 paradigm in allergy and autoimmunity has been revised, including the role of the Th17 cell population and related cytokines. However, there are only a few studies that have found Th17 cytokine involvement in the allergic inflammatory response, especially with food allergens. This research aimed to analyze the serum profile of cytokines involved in the T-helper cell type 17 immune response pathway in young, atopic children with an IgE-mediated and delayed-type FA. The study involved 76 children (0–5 years old) with chronic AD. We used the Bio-Plex system to simultaneously determine the concentrations of 15 different cytokines in one experiment. In accordance with complete dermatological and allergological examination, including OFC testing and ALEX2 assays, participants were divided into 3 groups: IgE-mediated FA, delayed-type FA, and the control group. Data were analyzed using univariate statistical tests. In the IgE-mediated FA group, the circulating levels of tested cytokines had increased compared with those of other patients; however, a statistically significant difference was only obtained for IL-1beta (*p* < 0.05). According to the ROC curves, IL-1beta may be considered an effective predictor of IgE-mediated FA in AD children (*p* < 0.05; AUC = 0.67). In the delayed-type FA group, the concentration of most cytokines had slightly decreased compared to the control group. The obtained results suggest that FA influences the Th17-related cytokine profile in the serum of AD children. More advanced studies are needed to confirm the involvement of Th17 cytokines in the allergic inflammatory response and to prove their usefulness in clinical practice.

## 1. Introduction

Allergic diseases constitute a huge challenge for contemporary medicine [1]. The seriousness of the problem is determined by the scale and complex pathogenesis of allergies. Epidemiological studies have confirmed that allergies are among the most common chronic diseases worldwide [2,3,4]. Putting aside the medical aspect, allergic conditions constitute social and economic problems, generating enormous costs and leading to a deterioration in the quality of patients’ lives [5,6]. In the face of this situation, the most important thing is to improve and optimize diagnostic and therapeutic procedures. This optimization is especially expected and desired in relation to food allergy (FA), the incidences of which have increased significantly over the last dozen or so years [7] and whose diagnostic methods are not fully reliable [8]. In vitro and in vivo tests can investigate IgE-mediated food hypersensitivity, yet there are no laboratory methods to diagnose other types of hypersensitivity, e.g., delayed-type FA [9]. The correlation between commercial assays and identifying allergen-specific IgE is poor (low sensitivity and specificity) [10]. The routine diagnostic methods that are currently performed are characterized by a high risk of false-negative and false-positive results [11]. The allergen extracts used in the in vitro and in vivo tests may have properties that have changed as a result of prior treatment when compared with the natural starting substrate [12]. In turn, naturally ingested food, as a result of digestion, can be a source of new allergenic molecules that induce the synthesis of specific IgE antibodies, which cannot be detected in standard assays [9,13]. Also, these problems are not solved by the new diagnostic systems that are based on component-resolved diagnosis (CRD), which have been used for several years [9,14,15]. In addition to this, currently, there is no effective cure for food hypersensitivities. Management of FA includes allergen avoidance or emergency treatment [16,17]. Accurate diagnosis of allergies in children is very important when assessing the risk of generalized reactions and the chance of developing a tolerance to a given allergen or the need to introduce dietary restrictions [18,19]. Therefore, there is still a need to search for new, more precise and accurate diagnostic methods, differentiating FA mechanisms so that a complete, personalized approach to the patient is possible.

The main risk factor for the development of FA is atopic dermatitis (AD) [20,21]. According to statistics, 30% of children who develop moderate to severe forms of AD additionally suffer from FA [22]. There is a hypothesis that states that the damaged skin barrier of eczema patients leads to the absorption of food allergens through the dermal coating and contributes to the development of sensitization [23,24,25]. Recent evidence shows that atopic lesions appear before the occurrence of sensitization to food ingredients and that FAs are most associated with a severe and chronic course of AD [24]. AD is usually recognized as the Th1/Th2 paradigm of allergic disease [26]. According to the literature, AD patients develop a biphasic immune response after exposure to allergens [27]. The first phase involves antigen presentation, induction of Th2 cells, and the release of IgE immunoglobulins. In the second phase of the disease, eosinophils are recruited and the phenotype of Th2 cells changes to the Th1 pathway [28]. The immune response pathway of acute AD lesions is almost analogous to that of IgE-mediated allergy and follows the Th2 pathway [29,30]. An analysis of cytokine expression showed that T helper cells of early lesions produce IL-4, IL-5, and IL-13, which are hallmark cytokines of Th2 cells [31,32]. In turn, chronic AD changes that are similar to delayed-type FA are manifested by Th1 cells [33,34,35,36]. Recently, the Th1/Th2 paradigm in allergy and autoimmunity has been revised, including the role of Th17 cell population [37]. The key mediators responsible for the involvement of naive Th cells in the Th17 phenotype, distinguishing Th17 cells from the Th1 and Th2 populations, are IL-1 beta, IL-23, TGF-beta, and IL-6 [31,38]. A hallmark of Th17 cells is the production of inflammatory cytokines such as IL-17A, IL-17F, IL-26, and IL-22 [39,40]. Th17 cells are involved in the development of autoimmune diseases, including rheumatoid arthritis (RA), multiple sclerosis (MS), and psoriasis, as well as having an involvement in the neutralization of extracellular pathogens such as Candida albicans and Mycobacterium tuberculosis [41]. In AD patients, IL-4 cytokine, produced by Th2 cells, strongly inhibits the immunity of Th17 and Th1 lymphocytes [42,43]. Some analyzes have even shown that IL-4 reduces the number of Th17 cells that induce and maintain cytokine IL-23 in antigen-presenting cells, both in vitro and in vivo in humans [44,45]. On the other hand, phenotypic analysis of peripheral blood mononuclear cells from severe AD patients confirmed a significant increase in the Th17 population compared to healthy volunteers, suggesting a direct relationship between the presence of Th17 cells and the severity of the disease [42]. It is still not fully understood why, despite Th2 cytokines (such as IL-4) suppressing IL-17 and IL-23, IL-17-producing cells are still detected in AD. Of note, Toda et al. [46] reported that IL-17 expression was much more evident in acute rather than in chronic lesions. The role of Th17 cells in allergy is still largely unresolved; there are no reliable studies that demonstrate the involvement of Th17 lymphocytes and related cytokines in allergic immune response, especially to food allergens. One of the few conducted studies suggested that peanut exposure increased Th17 and Th2 cytokine production in response to house dust mites in humanized mice that had been reconstituted with peripheral blood mononuclear cells from allergic donors [47]. Moreover, according to Khalid Bin Dhuban et al., no significant differences in Th17 cytokine concentration were observed between atopic and non-atopic individuals with FA [48]. This observation may suggest that FA mainly influences the Th17-related cytokine profile of atopic patients.

Therefore, this research aimed to characterize the serum profile of the cytokines involved in the T-helper cell type 17 immune response pathway in young, atopic children with IgE-mediated and delayed-type FA. Cytokines are important mediators of immunity, and their response due to an imbalance or deficiency in the cytokine network may largely determine immune disease susceptibility and severity [49]. Researchers have shown that interleukins, lymphokines, monokines, interferons (IFN), chemokines, and many other cytokines are involved in the allergic inflammatory response [50,51]. Recognition of changes in serum concentration of inflammatory factors will be the basis for further elucidation and differentiation of the molecular mechanisms of FA. Our analysis was performed using the Bio-Plex Pro Human Cytokine Assay (Bio-Rad, Hercules, CA, USA). The Bio-Plex multiplex immunoassay uses fluorescently colored magnetic beads to quantify a biologically relevant target. The innovative technology based on Luminex/xMAP allowed for the simultaneous determination of 15 different Th17-related cytokines in the serum of 76 children during one experiment.

## 2. Materials and Methods

The study was approved by the Bioethical Commission of Poznan University of Medical Sciences. All participants received an explanation of the main objectives and possible benefits of the study. Informed consent was obtained from all volunteers and, in the case of children, from their parents.

### 2.1. Study Groups

The scope of the research covered the diagnosis of major FAs in children aged 0 to 5 years. The study involved 76 volunteers, in whom there was a likely association between chronic symptoms of AD (L20) and exacerbations with food exposure. In addition, some patients developed acute symptoms following food exposure in the form of urticaria (L50), angioedema (T78.3), and/or anaphylaxis (T78.0). The complete characteristics of the demographic profile of patients are presented in Table 1.

All participants underwent a detailed medical examination, and their parents were obligated to carefully fill in a questionnaire. The information contained in the questionnaire allowed for the inclusion/exclusion of patients from the study and helped to assign them to the appropriate group. The criteria for including participants in the study and the rationale for selecting the group of children aged 0 to 5 are presented in Table 2.

After the patients’ parents or legal guardians were acquainted with the nature of the research and consented to participate in the study, venous blood was collected from all children. The serum samples for testing were collected at the Individual Specialist Medical Practice of MD Joanna Matysiak in Kalisz, and then stored at −80 °C until analysis.

A complete dermatological and allergological examination of the participants, including the examination of the sensitization to molecular components and extracted “whole” allergens, was performed using ALEX2 Allergy Explorer, Polycheck Allergy Diagnostics Multiparameter Technology (Pediatric 30-I) and singleplex ImmunoCAP testing. Based on the results of allergological in vitro tests, medical history, and oral food challenge tests (OFC), the participants were divided into target groups. The study group contained 36 young children suffering from AD and diagnosed with IgE-mediated FA (medical history +; sIgE +; OFC +), while the control group consisted of 40 atopic patients without IgE-mediated FA (medical history +/−; sIgE −; OFC +/−). In the next stage of the experiment, based on clinical symptoms and the OFC test, from the group of patients with negative sIgE results, we distinguished 15 children with delayed-type FA (medical history +; sIgE −; OFC +) and 25 participants without FA (medical history +/−; sIgE −; OFC −). The sera of all patients were subjected to Bio-Plex examination to characterize the pathophysiological conditions associated with IgE-mediated FA and delayed-type food hypersensitivity.

### 2.2. Diagnosis of IgE-Mediated Food Allergy

We examined IgE-mediated FA in the Polish population of young children with chronic symptoms of AD, determining the concentration of IgE antibodies specific to 330 allergens, concerning both the extracted “whole” allergens and the molecular components. We used the Allergy Explorer ALEX 2 test (MacroArray Diagnostics, Wien, Austria): a macroarray containing 295 allergens, the Polycheck Pediatric 30-I test (Biocheck, Germany): a screening panel of the 30 most common pediatric allergens, and singleplex ImmunoCAP assays (Thermo Fisher Scientific/Phadia, Uppsala, Sweden). In this experiment, the ImmunoCAP system was used to determine the level of IgE antibodies that were specific to 5 allergens: cow’s milk extract and its components, i.e., alpha-lactalbumin, beta-lactoglobulin, casein, and Bovine serum albumin.

**Figure 1:**
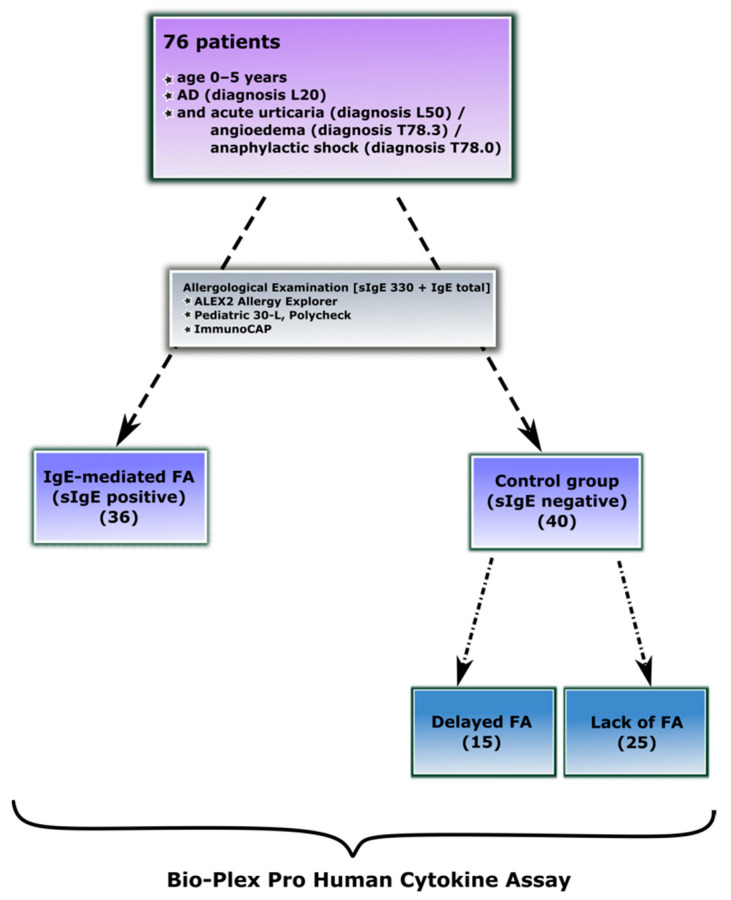
Graphical presentation of the study design with the division of participants into research groups.

The positive results of the allergen-specific IgE assays were confirmed by the OFC test. Based on physical examination and clinical history, the patients were subjected to the OFC test selectively. Children with a history of anaphylaxis and detectable levels of IgE specific to a suspected food were excluded from being tested with the oral food challenge. The conducted analyzes allowed for the division of participants into appropriate groups (Figure 1).

The principle of all in vitro analyzes was based on the ELISA method. The tested allergens were immobilized in a solid phase made of a 3D cellulose polymer. Cellulose-immobilized antigens reacted with specific antibodies present in the serum of patients. Unbound, free antibodies remained flushed away, thus preventing non-specific binding. Antibodies labeled with enzymes directed against the specific IgE were added sequentially. After the incubation step, the unbound p-sIgE antibodies were washed away. Then, an incubation step with the appropriate substrate was performed. The antibody-conjugated enzyme (peroxidase/alkaline phosphatase) was reacted with the colorless substrate to form a colored product. Finally, the degree of fluorescence of the enzymatic reaction product was measured. The color intensity/optical density was measured at 450 nm. The intensity of the color indicated the amount of the allergen-specific antibodies present [52].

### 2.3. Diagnosis of Delayed-Type Food Allergy

Diagnosis of delayed-type FA was based on observations of allergic symptoms after the intake of the suspected food. The most reliable clinical procedure for diagnosing IgE-mediated FA and non-IgE mediated FA is the oral food challenge test. The OFC comprises an oral administration of the suspected allergen in a controlled and standardized setting [53,54].

According to procedure, the suspected food allergen, e.g., cow’s milk protein, was completely excluded from the patient’s diet for a period of 4–6 weeks. Then, the parents of the patients were instructed in detail on how to reintroduce the previously excluded allergen into the patient’s diet. The diet extension continued until the allergy symptoms recurred or until the suspected food allergen was completely introduced, which confirmed or ruled out delayed-type FA. During the next follow-up visit, a detailed clinical interview was conducted, and a physical examination of the patient was performed. During the OFC, the suspected food allergen was administered daily, in gradually increasing doses.

Children undergoing the OFC had no history of anaphylaxis, no detectable high levels of IgE specific to suspected foods, no conditions that might affect their safety, and were not taking drugs, which might interfere with the assessment or affect safety.

### 2.4. Measurement of Th17 Cytokine Panel

Using the Bio-Plex Pro Human Th17 Cytokine Assay (Bio-Rad, Hercules, CA, USA), one experiment evaluated a comprehensive mix of fifteen pro-inflammatory factors that are involved in the T-helper cell type 17 immune response pathway (IL-1β, IL-4, IL-6, IL-10, IL-17A, IL-17F, IL-21, IL-22, IL-23, IL-25, IL-31, IL-33, IFN-ϒ, sCD40L, and TNF-α) according to the manufacturer’s instruction. The xMAP technology used in the study is based on three basic elements: fluorescently dyed microspheres/magnetic separation, a dedicated flow cytometer, and a high-speed digital signal processor. The kit consists of ready-to-use reagents, a 96-well plate, standards, and quality controls recommended for the assay. The Bio-Plex assay is essentially an immunoassay formatted on magnetic beads. The assay principle is similar to that of a sandwich ELISA. In simple terms, 50 µL of serum, standards, and quality control samples were added to consecutive wells containing antibody-conjugated magnetic beads, and then the mixture was incubated for one hour at room temperature. After the incubation and washing steps were completed, the biotinylated detection antibody was added to the magnetic beads to form a sandwich complex. The final reaction mixture was obtained by adding the fluorescent streptavidin-phycoerythrin conjugate. Cytokine concentrations were determined by flow cytometry using a Bio-Plex Matrix Reader (Bio-Plex MAGPIX, Bio-Rad, Hercules, CA, USA), which is equipped with two LEDs; the first emits red light with a wavelength of 635 nm, while the light emitted by the second diode is green with a wavelength of 532 nm. Data acquisition was performed using Bio Plex Manager 6.0 software (Bio-Rad, Hercules, CA, USA). Before the final analysis, the software was verified and fully calibrated. The standard curve was designed based on the standards provided by the manufacturer. The concentrations of the tested factors were presented in picograms per milliliter (pg/mL) based on the standard curves. Two of the ninety-six wells were filled with Bio-Rad diluents and considered as blank for the experiment. In order to verify the correctness of the analysis, quality controls (low and high level) were used.

### 2.5. Data Analysis

Statistica 13.0 (StatSoft Inc., Tulsa, OK, USA) and MedCalc (MedCalc Software Ltd., Ostend, Belgium) were used to conduct statistical analyzes. A numerical value of *p* < 0.05 was considered statistically significant. Data were analyzed using univariate statistical tests. Depending on the type of data distribution between the study group and the control group, the obtained values were compared using the Mann-Whitney test, the Student’s t-test or the Welch test. The Shapiro-Wilk test was used to check the normality of the distribution of the results. Variables with a normal distribution had their equality of variance tested with Levene’s test, while variables without a normal distribution were compared using the Mann-Whitney U test. If Levene’s test results were not statistically significant (*p* > 0.05), a Student’s t-test was conducted. The Welch test was applied if Levene’s test results were statistically significant (*p* < 0.05). Additionally, the Kruskal-Wallis one-way ANOVA test was used to compare the data between the three groups. A univariate receiver operating characteristic (ROC) curve was calculated using the MedCalc software (MedCalc Software Ltd., Ostend, Belgium). For each tested analyte, a standard univariate ROC curve was calculated to graphically present the correlation between sensitivity and specificity.

## 3. Results

The adoption of a novel methodology based on the Luminex/xMAP technology allowed for the simultaneous determination of several markers of the inflammatory process in the serum during one experiment. We successfully measured the serum concentrations of nine out of the fifteen determined cytokines (IL-1beta, IL-4, IL-17A, IL-22, IL-23, IL-25, IL-31, sCD40L, and TNF-alpha) from seventy-five out of the seventy-six patients. The values for the six analyzed proteins were below the detection level for all analyzed samples or were only detected in parts of the samples. These cytokines were excluded from the data analysis. Finally, the serum concentrations of the nine cytokines from seventy-five patients were statistically analyzed.

### 3.1. Alterations in Serum Concentration of the Th17-Related Cytokines

#### 3.1.1. Comparison of Serum Cytokine Concentrations between the Allergy Group (1) and the Control Group (0)

**Figure 2:**
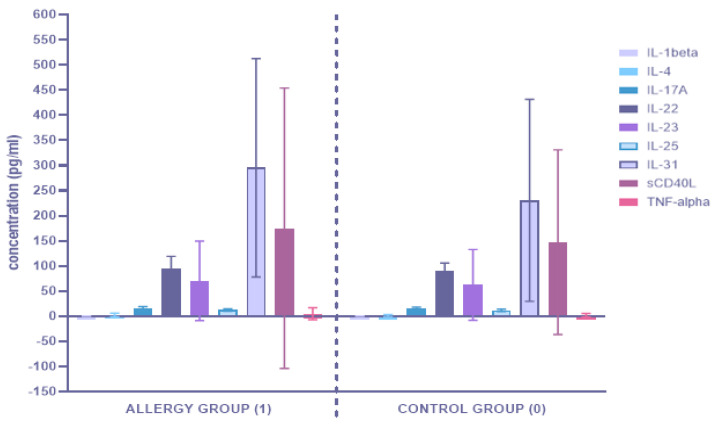
Comparison of serum cytokine concentrations between the allergy group (1) and the control group (0).

The concentrations of inflammatory factors in the serum of 35 patients with IgE-mediated hypersensitivity (allergy group (1)) and 40 patients without IgE-mediated FA (control group (0)) were measured. The determined concentration values are presented in Figure 2 and Figure S1, Table S1.

The inflammation profile of the AD patients diagnosed with IgE-mediated hypersensitivity (allergy group (1)) was compared to those without IgE-mediated FA (control group (0)) using a U Mann-Whitney test. The results of the statistical analysis are presented in Table 3. The univariate statistics showed that statistically significant differences among the analyzed groups occurred for the concentration of one cytokine. In the allergy group, the circulating concentration of interleukin 1 beta had significantly increased (*p* < 0.05) compared to that in the control group. Similar differences were observed in the levels of other inflammatory factors between the study groups, although these differences were not statistically significant.

#### 3.1.2. Comparison of Serum Cytokine Concentrations between the Three Studied Groups: The IgE-Mediated Allergy Group (1), the Delayed-Type Allergy Group (2), and the Control Group (3)

**Figure 3:**
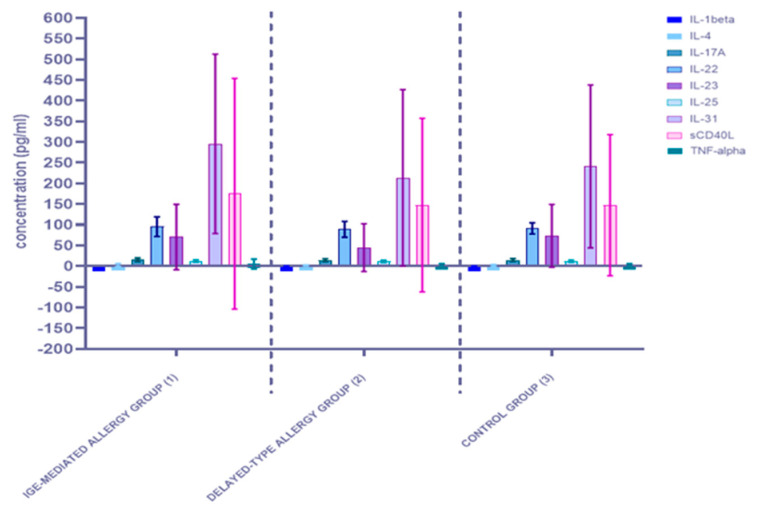
Comparison of serum cytokine concentrations between the three studied groups: the IgE-mediated allergy group (1), the delayed-type allergy group (2), and the control group (3).

In the next stage of the experiment, based on the results of allergological in vitro examination, medical history, clinical symptoms and OFC testing, all participants were divided into three groups: the IgE-mediated FA (35 patients); the delayed-type FA (15 patients); the control group (25 participants). The measured cytokine concentrations for each group are shown in Figure 3 and Figure S2, Table S2. The mean concentrations of all nine studied inflammatory factors had increased for the IgE-mediated FA group compared to the rest. In the group of patients diagnosed with delayed-type FA, only the mean concentration of IL-1 beta had increased when compared to the control group.

To compare the results between the three groups, a Kruskal-Wallis one-way ANOVA was performed. Statistically significant differences occurred in the concentration of one inflammatory marker. The concentration of IL-1beta was significantly (*p* < 0.05) higher in the IgE-mediated allergy group when compared to the other groups. In turn, among patients with delayed-type hypersensitivity, the level of interleukin 1 beta was significantly (*p* < 0,05) higher when compared to the control group and significantly (*p* < 0.05) lower in comparison with IgE-mediated allergy patients. More detailed results are presented in Table 4, with the statistically significant value in bold and underlined text. The concentrations of other cytokines in all analyzed groups differed from each other, however, these differences were not statistically significant.

### 3.2. Usefulness of the Th17-Related Cytokines in the Differentiation of Food Hypersensitivities in Children with Atopic Dermatitis

**Figure 4:**
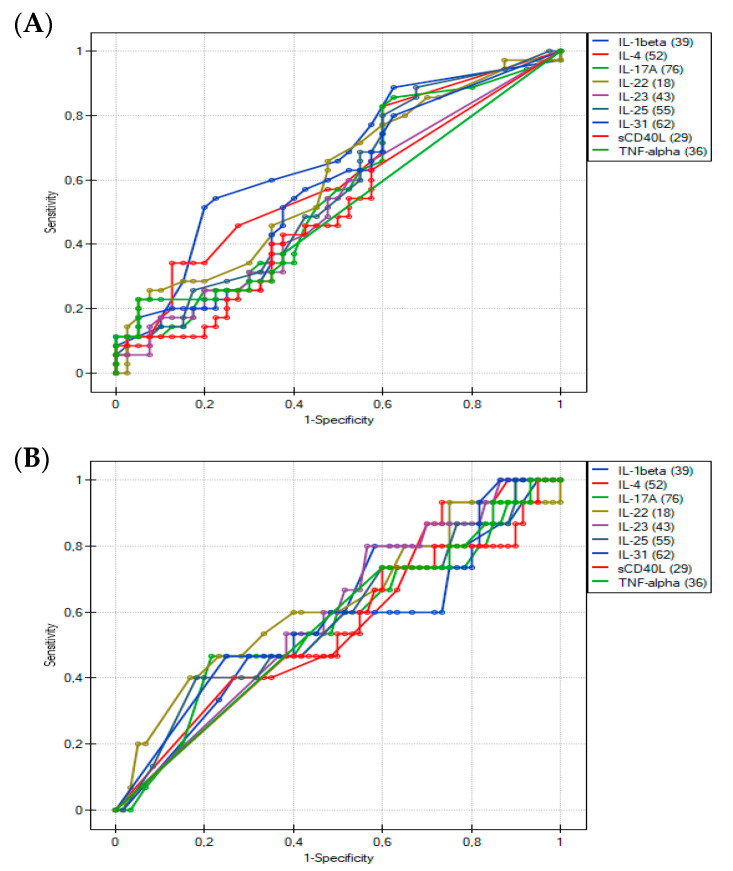
Univariate receiver operating characteristic (ROC) curves show a correlation between the serum concentrations of nine cytokines (**A**) in patients with IgE-mediated allergy and other participants; (**B**) in patients with delayed-type food allergy and other participants.

**Figure 5:**
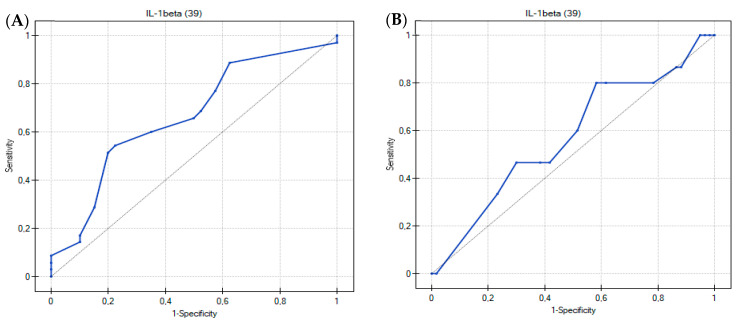
Univariate receiver operating characteristic (ROC) curve shows the correlation between the serum concentration of IL-1 beta (**A**) in patients with IgE-mediated allergy and other participants; (**B**) in patients with delayed-type food allergy and other participants.

The discriminating ability of Th17 cytokines was further tested by calculating ROC curves (Figure 4), which summarize and give a graphical presentation of the sensitivity and specificity of the studied markers. In our study, the area under the curve of approximately 0.7 was found to be satisfactory. Based on the obtained graphs, interleukin 1 beta can be considered as a potentially differentiating factor of IgE-mediated FA in young children with chronic symptoms of AD (Figure 4A and Figure 5A). The highest AUC value (0.67) obtained was for IL-1 beta, with a specificity of 77.5% and a sensitivity of 54.3% at a cut-off value of 0.494 pg/mL (Table 5). On the other hand, a comparison of the ROC curves for IL-1 beta between the study groups showed that IL-1 beta cannot be considered as a prognostic factor for type IV hypersensitivity (Figure 4B and Figure 5B). The results of the ROC curves analysis for the remaining cytokines were also unsatisfactory (*p* > 0.05; AUC < 0.65) (Table 5).

## 4. Discussion

In this study, we focused on the analysis of the serum profile of inflammatory factors in young children with food allergy (FA) and chronic symptoms of atopic dermatitis (AD). We successfully measured the concentration of nine cytokines involved in the T-helper cell type 17 immune response pathway: IL-1 beta, IL-4, IL-17A, IL-22, IL-23, IL-25, IL-31, sCD40L, and TNF-alpha. In one experiment, we examined several serum inflammatory markers using the Bio-Plex method (Bio-Rad). The Bio-Plex system utilizes xMAP technology to enable the multiplexing of up to 100 different analytes [55]. Multiplexed analysis has the advantage of simultaneously detecting multiple analytes in a single reaction vessel reducing time, labor, and cost when compared to single-reaction-based detection methods. The Luminex/xMAP system offers high-throughput detection of target proteins. It has been used in a variety of applications ranging from biomarker discovery and validation, pathogen detection, drug discovery, vaccine development, and cancer research [56]. Previously, using the Bio-Plex system, Bingtai Lu et al. measured the concentration of cytokines in the plasma and bronchoalveolar lavage supernatant of children with community-acquired pneumonia [57], while Bouadma L et al. assessed the immune alterations in a patient with SARS-CoV-2-related acute respiratory distress syndrome [58]. Our study compared the inflammation profiles of AD patients diagnosed with IgE-mediated FA, delayed-type FA, and AD children without FA. As the results show, in the IgE-mediated allergy group, serum concentrations of the analyzed cytokines involved in the Th17 pathway were higher than those of other patients; however, a statistically significant difference was only obtained for IL-1 beta (*p* < 0.05). Among patients with delayed-type hypersensitivity, the concentrations of most cytokines were slightly lower, while the level of interleukin 1 beta was significantly (*p* < 0.05) higher compared to the control group and significantly (*p* < 0.05) lower in comparison with IgE-mediated allergy patients. Based on the univariate ROC curve, which graphically shows the sensitivity and specificity of the analyzed marker, interleukin 1 beta can be considered a prognostic factor for IgE-mediated FA.

**Figure 6:**
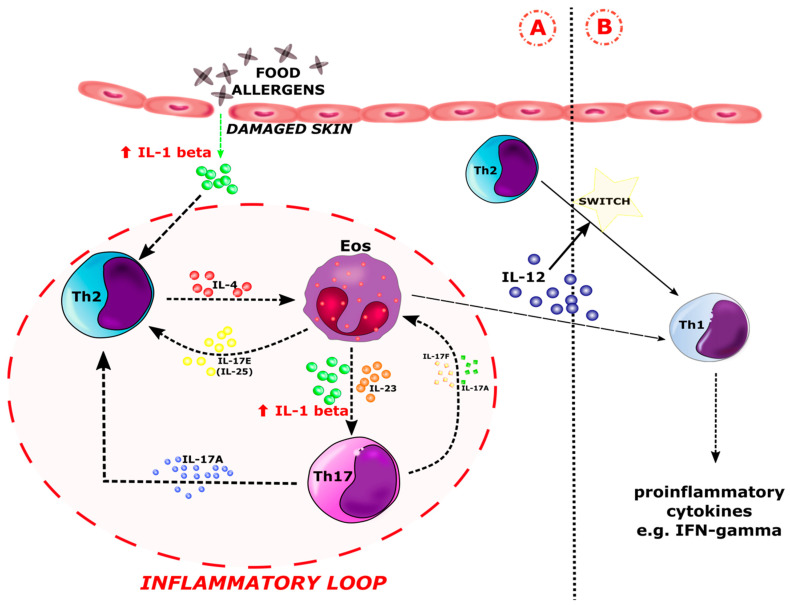
Involvement of IL-1 beta in the inflammatory response in patients with food allergy (FA) and atopic dermatitis (AD). There is an increased concentration of IL-1 beta in (**A**) IgE-mediated hypersensitivity along the Th2 pathway, similar to the acute phase of AD. There are two possible sources of IL-1 beta. The first is damaged skin. In response to the contact of food allergens with damaged tissue, IL-1 beta is released, leading to the increased production of Th2 cells. On the other hand, IL-1 beta is produced and released by recruited eosinophils that migrate to inflammatory sites in response to type 2 cytokines, e.g., IL-4 [89]. Increased concentration of IL-1 beta leads to the polarization of naive Th cells into the Th17 lineage. The resulting Th17 lymphocytes release the cytokines IL-17A and IL-17F, which affect both the Th2 cells and the eosinophils. In addition to IL-1 beta, eosinophils release, among others, IL-25 (IL-17E), which enhances the production of Th2 inflammatory mediators. The interaction of Th2 cells, eosinophils, and Th17 via their cytokines drives the inflammatory loop. The inflammatory loop is marked with a dashed red line. IL-1 beta is the major initiator of the inflammatory loop cascade (**A**). Otherwise, eosinophils are able to switch between the Th2 and Th1 pathway via IL-12. It is the site of transition for (**B**) delayed food allergy along the Th1 pathway, similar to chronic AD. Eos–eosinophil; Th2–type 2 helper T cell; Th17–type 17 helper T cell; Th1–type 1 helper T cell.

Interleukin-1 beta (IL-1 beta) is a highly pro-inflammatory cytokine expressed in antigen-presenting cells (APC) and was discovered and first described as a lymphocyte activating factor [59,60] or leukocytic pyrogen [61] in the 1970s. IL-1 beta has a multidirectional action. It stimulates B lymphocytes to proliferate and produce IgE antibodies, promoting the survival of T lymphocytes and enhancing the polarization of naive Th cells towards the Th17 phenotype [62]. IL-1 beta plays an important role in the innate immune response, initiating an acute response to infection and injury. Its elevated levels have been observed in chronic inflammation. According to literature reports, administration of IL-1 beta to patients in the context of haematopoietic stimulation led to adverse effects such as fevers, muscle, head and joint pain, fatigue, neutrophilia, and increases in acute phase proteins [63,64,65]. Therefore, it is considered to be a mediator that leads to a significant increase in the inflammatory process. IL-1 beta is involved in the development of common diseases such as type II diabetes [66], gout [67], and cancer [68]. It is responsible for the formation of urticarial rash in autoinflammatory diseases [69] and is involved in the pathogenesis of contact hypersensitivity, atopic dermatitis [70,71], asthma [72,73,74], and rheumatoid arthritis (RA) [75]. Nutan FN et al. noted a high level of IL-1 beta in the serum of AD patients. According to their research, IL-1 beta levels were raised during disease activity and corresponded with the severity of AD [76]. Our analysis showed significantly (*p* < 0.05) elevated levels of IL-1 beta in AD patients diagnosed with IgE-mediated FA compared to AD patients with delayed FA and AD participants without FA. We also observed a slightly increased concentration of this interleukin in the group of atopic patients with non-IgE-mediated allergy in comparison with the atopic children without allergies. According to our data, elevated levels of IL-1 beta in atopic children diagnosed with IgE-mediated FA suggest its involvement in the development of an allergic inflammatory response to food allergens, which may lead to an exacerbation of AD. There is a hypothesis that states that exposure to the allergen through the skin causes the release of IL-1 beta from damaged tissues, contributing to the production of Th2 cells [77]. Assessment of cytokine expression indicated that Th2 cells release IL-4 and IL-5, which are responsible for the recruitment of eosinophils to inflammatory skin sites during early lesions of AD [78]. At the same time, memory T cells are redistributed into the bloodstream and can infiltrate skin tissues, leading to an exacerbation of AD. In addition, T cells spread beyond the skin and initiate an atopic march, including FA. Eosinophils that arrive at inflammatory sites begin to produce IL-1beta, IL-23, and IL-17E. Interleukin 17E influences the number of Th2 cells, releasing many cytokines. On the other hand, IL-1beta and IL-23 drive Th17 cell polarization [28]. Cheungi et al. suggested that IL-17A and IL-17F may promote the formation of eosinophils, as well as increase the levels of IL-1beta and IL-23 [79]. These findings support the interaction between eosinophils and Th17 cells in the pathogenesis of atopic disorders. Esnault et al. showed that an increase in IL-17 expression in Th17 cells occurred as a response to IL-1 beta being released by eosinophils [80]. Eosinophils have been reported to be a source of IL-1 beta and express the active protease of interleukin-1 beta converting enzymes (ICE) [81,82,83,84]. The presence of eosinophils in the inflammatory infiltrate within allergic conditions has been confirmed. Most often, the severe form of atopic disease correlates with an increased number of eosinophils and an increased release of eosinophil-derived proteins, including IL-1 beta [85]. In patients suffering from both AD and FA, eosinophil recruitment to the inflammatory sites is probably enhanced due to the increased secretion of Th2/Th17 cytokines during the overlapping of these diseases. Th2 cytokines such as IL-4, IL-13, and IL-9 promote eosinophilia by regulating local IL-5, eotaxin synthesis, and/or inhibiting IFN-gamma production [85]. On the other hand, IL-4 reduces the level of IL-23, which, similar to IL-1 beta, is synthesized by the eosinophils and induces and maintains Th17 cells. Koga et al. demonstrated a lower expression of IL-17 in the chronic phase of AD in comparison to acute atopic lesions [28,42,46]. These observations support our results that in patients with chronic AD, IgE-mediated FA mainly influences the serum profile of cytokines involved in the Th17 immune response pathway by enhancing their expression. Lower levels of IL-1 beta have probably been observed in patients with delayed-type FA because, like chronic AD, Th1 cells mainly drive its pathogenesis. It should be also noted that activated eosinophils produce matrix metalloproteinase-9 (MMP-9), leading to the independent release of IL-1 beta by the inflammasome/caspase-1 [86]. The induction of IL-1 beta expression occurs in the absence of additional signals, independent of the presence of other cytokines, which may explain its elevated level in the acute phase of AD and IgE-mediated allergy. Eosinophilic IL-1 beta modulates the course of the inflammation and may be a link between the Th2 and Th17 immune response pathways [87]. On the other hand, eosinophils may be responsible for switching the Th2-dominated immune response to a Th1 phenotype in chronic atopic lesions and delayed-type FA via IL-12 production [88]. Based on our results and data from the literature, we proposed a scheme for the involvement of Il-1 beta in the pathogenesis of FA in patients suffering from AD (Figure 6). The presence of Th1-, Th2-, and Th17-related cytokines, the overproduction of IgE immunoglobulins, and the recruitment of eosinophils to inflammatory sites, underlines the effector pathway common to AD and FA, which may partially explain their selected clinical and pathological similarities.

**Figure 7:**
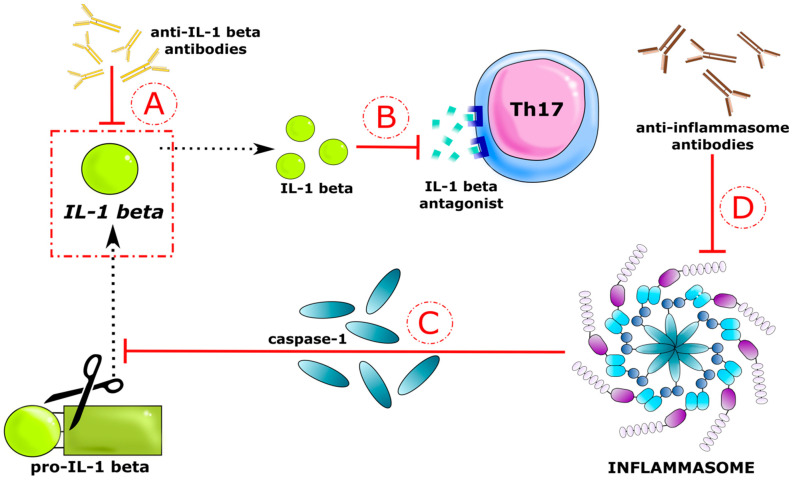
IL-1 beta as a therapeutic target in reducing the allergic inflammatory response. The red arrows represent the likely therapeutic impact sites. IL-1 beta is a highly pro-inflammatory cytokine that has been found to increase in children with IgE-mediated food allergy (FA) and atopic dermatitis (AD). IL-1 beta secreted by cells in an inactive form as pro-IL-1 beta is activated by caspase-1 released from the inflammasome. It acts through two receptors to trigger the polarization of naive Th cells into the Th17 lineage. We propose four therapeutic approaches at each stage of IL-1 beta formation and an action to reduce the inflammatory response. (**A**) Direct blockage of IL-1 beta by anti-IL-1 beta antibodies; (**B**) blockage of the IL-1 beta receptor by the antagonist; (**C**) neutralization of caspase-1, which is the pro-IL-1 beta activating enzyme; (**D**) inactivation of inflammasomes.

In the development of allergic disorders, a special role is ascribed to immunological processes. In the future, the inhibition of the activity of pro-inflammatory cytokines may be helpful in treating children with chronic AD and IgE-mediated FAs. Nowadays, some inflammatory diseases are treated by blocking IL-1 beta [90]. IL-1 beta is not found in the cells of healthy individuals and requires a series of intracellular events before it can cause inflammation. It is synthesized and secreted in response to contact with an inflammatory stimulus as an inactive cytoplasmic precursor (pro-IL-1 beta). Inactive IL-1 beta accumulates in the cytoplasm and undergoes the process of splitting into active proteins in the presence of inflammasomes [91]. Activation of the inflammasome triggers the conversion of the caspase-1 precursor to its active form, which in turn cleaves the inactive form of IL-1 beta [62]. Interleukin 1 beta signals through two receptors: IL-1RI and IL-1RII, and its activity can be suppressed by an IL-1RA receptor antagonist that blocks cytokine binding to the receptor through competitive inhibition. IL-1 neutralizing drugs have been shown to completely suppress or significantly reduce inflammatory responses in clinical studies and experimental models of urticarial autoinflammatory diseases as well as common allergic disorders [92]. In a study by Esnault et al., IL-1 beta neutralizing antibodies reduced the expression of IL-17 mRNA. The IL-23/IL-17 axis is an important pathway in the targeted therapy of inflammatory diseases [80]. Emerging evidence from clinical trials has shown that monoclonal antibodies to IL-23, IL-17, and TNF are effective in treating patients with psoriasis, atopic dermatitis, pyoderma, rosacea, pemphigus, and scleroderma systemic [80]. Currently, there is still no research on the use of such therapeutic agents in FAs. Accordingly, we would like to propose a mechanism involving interleukin 1 beta by which the inflammatory response in allergic/atopic diseases can be reduced (Figure 7).

In this study, we observed elevated levels of the remaining eight cytokines (IL-4, IL-17A, IL-22, IL, 23, IL-25, IL-31, sCD40L, and TNF-alpha) in the serum of patients with IgE-mediated hypersensitivity in comparison to other participants; however, the obtained differences were not statistically significant. According to data from the literature, most of these inflammatory factors are involved in the course of allergic conditions [93]. IL-4 and IL-31 mediate the activation of eosinophils and mast cells and indirectly activate B lymphocytes to produce IgE, contributing to skin itching and allergic inflammation [94,95]. IL-4 is the major cytokine involved in the initiation of the Th2 response [94]. IL-23 is involved in the activation of Th17 cells to induce the production of IL-17A, IL-17F, TNF, and IL-6 [96]. Many studies have shown the role of IL-17A in the pathogenesis of allergic disorders, including food hypersensitivity [97,98]. However, to date, most studies on the role of IL-17A in the pathogenesis of allergic diseases have been carried out using animal models, so it is difficult to apply the obtained conclusions to humans. In the course of AD, an elevation of the transcript of another member of IL-17 family (IL-17E) was found [99]. Yesilova et al. indicated high levels of TNF-alpha in children with atopic lesions [100]. TNF-alpha released from mast cells stimulates the state of allergic inflammation as it induces the production of Th2 cytokines and increases the migration of mediators to the inflammatory region [101]. Kara et al. concluded that TNF-alpha may be a useful marker for monitoring food hypersensitivities, especially those mediated by immunoglobulin E [102]. Our data show that the concentration of TNF-alpha was increased in patients with IgE-mediated allergy and slightly decreased in participants with delayed-type hypersensitivity when compared to the control group. The mechanism by which the expression of this cytokine decreases remains unknown and requires further investigation. We can only presume that disease conditions mediated by an independent Th1 pathway, such as delayed-type FA, will show a lower expression of Th17- and Th2-related cytokines.

This is the first study to use Bio-Plex to determine the serum concentration of multiple inflammatory cytokines involved in the T-helper cell type 17 immune response pathway in atopic children with food hypersensitivity, making it impossible to accurately compare the results with other researchers. We assume that the identification of changes in serum concentration (of factors involved in the allergic inflammatory response) constitutes the basis for further clarification and differentiation of the molecular mechanisms of FAs. Currently, there is no specific biological indicator for IgE-mediated and delayed-type FA in patients with chronic symptoms of AD. According to the literature, total serum IgE level is not a specific marker of IgE-mediated allergies [103]. A breakthrough in the history of FA diagnostics saw the development of component-resolved diagnosis (CRD), related to genetic engineering [14]. Molecular techniques allow for a more precise diagnosis of sensitization and create the possibility of personalizing therapeutic recommendations. The use of CRD significantly increases the specificity of the in vitro tests by assessing the primary sensitization and simultaneously enhancing diagnostic sensitivity, especially in a situation where clinically significant molecules are in the minority or absent in the “whole” allergen extract [14]. However, CRD is only applicable to type I hypersensitivity, which is connected to the production of IgE antibodies reacting with allergen epitopes [104]. Before using advanced and expensive tools of CRD, e.g., ALEX tests and ISAC assays, it should be possible to distinguish the mechanism of FAs. At present, there is no precise specific biomarker that differentiates IgE-mediated FA and delayed-type hypersensitivity. The results of this study provide a fresh and interesting look at the process of sensitization to food allergens in the course of AD. Univariate ROC curves show that IL-1 beta may help predict IgE-mediated FA in the course of AD. According to the literature, changes in metabolome and proteome may play a prognostic role, and these appear earlier than allergen-specific antibodies. Earlier diagnosis of FA is especially important in young children suffering from AD. The course and effectiveness of treatment can be improved after identifying and eliminating the allergenic factor [22,24]. In older patients, it is likely that other comorbid inflammatory and autoimmune diseases may also affect IL-1 beta expression [66,69,70,73,75]. Nevertheless, our study is the first step in finding a set of several proteins and metabolites that together will specifically differentiate the types of food hypersensitivity. Additionally, we may consider an IL-1beta receptor antagonist analog as a potential therapeutic agent for reducing the inflammatory response in patients with IgE-mediated FA and AD. IL-1RA can block the binding of IL-1 beta and thus inhibit the immune cascade, mainly related to the Th17 response [62]. Circulating levels of proteins are dynamic and modifiable and therefore amenable to therapeutic targeting. However, at the moment, these are only our assumptions which would require a confirmation from more advanced, large-scale research. Our study had some limitations. The obtained results are of a cognitive nature and expand the understanding of the role of cytokines in the allergic inflammatory response to food, yet currently are not useful for clinical practice. Moreover, we only analyzed the serum profile of cytokines related to the Th17 immune response pathway and did not examine the number, and contributions, of Th17 cells in the pathogenesis of AD and FA. Therefore, many aspects still remain unclear and unexplained.

In the future, identification of the presence of both Th17 cells and Th17-related cytokines in the course of FA and AD may reveal a common effector pathway for these diseases. AD appears to precede and trigger the development of FA in young children [24]. Nevertheless, further studies are needed to explain the common molecular mechanisms of these disorders. Getting to know them may contribute to improving the quality of life of young patients by limiting or even withdrawing the atopic march.

## 5. Conclusions

Cytokines are critical in allergic intercellular communication networks and contribute to disease pathology by recruiting and activating pro-inflammatory leukocytes. According to our results, cytokines involved in the T-helper cell type 17 immune response pathway can contribute to the pathogenesis of classically recognized Th2-mediated FA. Increased serum concentrations of IL-1 beta may suggest the development of IgE-mediated FA in young children suffering from chronic AD without additional comorbidities. In the future, there is a great need to investigate the involvement of both Th17 cells and Th17-related cytokines in the allergic inflammatory response and demonstrate their usefulness in clinical practice. Our results are the basis for further, more advanced analysis of allergy mechanisms and the search for new therapeutic targets and markers, differentiating the types of food hypersensitivity at the proteomic level.

## Figures and Tables

**Table 1:** Characteristics of participants.

Characteristics of the Participants	IgE-Mediated Food Allergy (1)	Control Group Lack of IgE-Mediated Food Allergy (0)	
		Delayed-Type Food Allergy (2)	Control Group Lack of Food Allergy (3)
**No. of subjects**	36	15	25
**Sex**			
**Male**	19 (52.8%)	11 (73.3%)	14 (56%)
**Female**	17 (47.2%)	4 (26.7%)	11 (44%)
**Age [months]**			
**Median**	23.5	12	20.5
**Mean**	25.81	13.2	26.6
**Range**	6–60	6–36	2–60
**Eczema [for the last 6 weeks or more]**	36	15	25
**Patient age on the onset of eczema [months]**			
**Median**	3	3	4
**Mean**	4.4	3.73	8.9
**Range**	1–24	1–10	2–36
**Atopic dermatitis (L20)**	36	15	25
**Association with foods:**			
**Milk**	27	11	13
**Egg**	9	5	2
**Egg white**	1	1	0
**Egg yolk**	1	0	0
**Cocoa**	5	0	3
**Chocolate**	2	0	0
**Oatmeal**	0	1	0
**Flour**	0	1	0
**Wheat**	1	1	3
**Gluten**	3	1	0
**Rye bread**	0	1	0
**Ketchup**	1	0	0
**Sweets**	1	0	0
**Nuts**	6	0	1
**Coconut**	2	0	0
**Fruits**	10	3	2
**Banana**	0	1	1
**Strawberry**	4	1	0
**Apple**	2	1	0
**Peach**	1	0	0
**Citrus**	2	0	1
**Juice**	0	0	2
**Carrot**	2	0	0
**Tomato**	1	0	0
**Potato**	1	0	0
**Soy**	0	1	0
**Chickpeas**	0	0	1
**Silage**	1	0	0
**Allergic Urticaria (L50)**	8	0	2
**Angiodema (T78.3)**	5	0	0
**The causative food:**			
**Hazelnut**	1	0	0
**Milk**	2	0	0
**Egg**	3	0	0
**Egg white**	1	0	0
**Peanut**	2	0	0
**White fish**	1	0	0
**Raisins**	1	0	0
**Gluten**	1	0	0
**Cauliflower**	0	0	1
**Anaphylactic shock (T78.0)**	5	0	0
**Symptoms of a generalized reaction:**			
**Dyspnoea**	5	0	0
**Vascular edema**	2	0	0
**Hives**	1	0	0
**Adrenaline**	0	0	0
**The causative food:**			
**Hazelnut**	1	0	0
**Milk**	1	0	0
**Peanut**	1	0	0
**Egg**	1	0	0
**Fish (cod)**	1	0	0
**Chronic symptoms of the digestive system:**			
**Colic**	0	6	0
**Abdominal pain**	2	3	0
**Abdominal gas**	1	2	0
**Vomiting, Downpouring**	0	3	0
**Diarrhea**	3	1	2
**Constipation**	2	1	0
**Mucus in the stool**	1	1	0
**Blood in the stool**	0	0	0
**Chronic diseases:**			
**Early childhood asthma**	3	2	1
**Recurrent bronchitis**	0	0	0
**Allergic rhinitis**	7	4	3
**Recurrent upper respiratory tract infections**	1	0	0
**Neurogenic bladder**			
	1	0	0

**Table 2:** Presentation of the main criteria for the inclusion of participants in the study and the justification for the selection of the research group (children 0–5 years old).

Main Criteria for Inclusion	Justification for the Selection of the Test Group
○ age 0–5 years	○ food allergy is one of the two earliest manifestations of an allergic disease (in the so-called allergy march)
○ (optional) episode of acute food allergic reaction: acute urticaria (diagnosis L50)/angioedema (diagnosis T78.3)/anaphylactic shock (diagnosis T78.0)	○ food allergies (mainly to cow’s milk proteins) gradually disappear in the following years of life (90% of children grow out of cow’s milk proteins allergy by 5 years of age)
○ chronic, recurrent skin lesions (for the last 6 weeks) in the course of atopic dermatitis (diagnosis L20)	○ the onset and most frequent occurrence of atopic dermatitis in the first years of life

**Table 3:** The results of univariate statistical analysis of the serum cytokine concentrations of patients with IgE-mediated allergy (allergy group (1)), and participants without IgE-mediated allergy (control group (0)).

Cytokine	*p* Value		
	Shapiro-Wilk Test		Mann-Whitney U Test
	Allergy Group (1)	Control Group (0)	
***IL-1beta***	0.079521	0.000159	0.014583
***IL-4***	<0.0001	<0.0001	0.087304
***IL-17A***	0.003265	0.008829	0.395553
***IL-22***	0.000282	0.012779	0.121022
***IL-23***	0.000128	<0.0001	0.659412
***IL-25***	0.281176	0.005543	0.274022
***IL-31***	0.016542	0.000028	0.260290
***sCD40L***	<0.0001	<0.0001	0.923855
***TNF-alpha***	<0.0001	<0.0001	0.815267

**Table 4:** The results of univariate statistical analysis of the serum cytokine concentrations of patients with IgE-mediated allergy (1), delayed-type allergy (2), and participants without food allergy (3). A statistically significant value (*p* < 0.05) is underlined.

Cytokine	*p* Value				
	Shapiro-Wilk Test/K-S			ANOVA Kruskal-Wallis Test	ANOVA Test
	IgE-Mediated Allergy Group (1)	Delayed-Type Allergy Group (2)	Control Group (3)		
***IL-1beta***	0.079521	0.024564	0.003197	0.0448	
***IL-4***	<0.0001	0.021308	0.000147	0.2189	
***IL-17A***	0.003265	0.042197	0.151014	0.6334	
***IL-22***	0.000282	0.014216	0.657141	0.2055	
***IL-23***	0.000128	0.002405	0.001771	0.5333	
***IL-25***	0.281176	0.061647	0.067417		0.3687
***IL-31***	0.016542	0.001127	0.003738	0.4952	
***sCD40L***	<0.0001	0.000361	0.000706	0.9312	
***TNF-alpha***	<0.0001	<0.0001	<0.0001	0.7305	

**Table 5:** The discriminant value of serum expression in all examined cytokines showing significant *p*-values and the area under the receiver operating characteristic (ROC) curve (AUC) between studied groups (the IgE- mediated FA group (1) vs. the control group (0)). The statistically significant value is in bold and underlined text.

*ROC Curve Analysis*	*Cytokines*								
	IL-1beta	IL-4	IL-17A	IL-22	IL-23	IL-25	IL-31	sCD40L	TNF-Alpha
***AUC***	0.67	0.62	0.56	0.61	0.53	0.57	0.58	0.51	0.52
***SE(AUC)***	0.063	0.065	0.067	0.066	0.066	0.067	0.066	0.066	0.060
***−95% CI***	0.540589	0.488565	0.425263	0.475555	0.401245	0.443203	0.446278	0.377998	0.399014
***+95% CI***	0.788697	0.742149	0.689737	0.733731	0.658755	0.704655	0.705864	0.635574	0.633129
***p value***	0.0144	0.0863	0.3926	0.1198	0.6556	0.2717	0.2580	0.9196	0.8111

## Data Availability

The data presented in this study are available in Supplementary File Spreadsheet S1: List of concentrations of 9 cytokines detected in all samples.

## Supplementary Materials

The following supporting information can be downloaded at: https://www.mdpi.com/article/10.3390/ijerph19137877/s1, Figure S1: Detailed comparison of serum cytokine concentrations between the allergy group (1) and the control group (0); Figure S2: Detailed comparison of serum cytokine concentrations between the three studied groups: the IgE-mediated allergy group (1), the delayed-type allergy group (2), and the control group (3); Table S1: Concentration values of nine cytokines in the allergy (1) and control (0) groups. The numerical variables are given in pg/mL; Table S2: Concentration values of nine cytokines in the IgE-mediated allergy group (1), the delayed-type allergy group (2), and the control group (3). The numerical variables are given in pg/mL.
