# Supplementary material for: Analysis of the Serum Profile of Cytokines Involved in the T-Helper Cell Type 17 Immune Response Pathway in Atopic Children with Food Allergy
Source: Int J Environ Res Public Health. 2022 Jun 27;19(13):7877. doi: 10.3390/ijerph19137877 (PMC9265836; doi:10.3390/ijerph19137877)
Supplement: Supplementary file 1 [file ijerph-19-07877-s001.zip › Supplementary Materials.pdf]

Supplementary Materials:

Table S1. Concentration values of nine cytokines in the allergy (1) and control (0) groups. The numerical variables are given in pg/ml.

Cytokine	ALLERGY GROUP (1)			CONTROL GROUP (0)		
	Median	*Mean*	*SD*	*Median*	*Mean*	*SD*
IL-1beta	0,5136	0,4811	0,1160	0,4265	0,4184	0,0944
IL-4	2,0702	2,9160	3,5823	1,5896	1,7493	2,0016
IL-17A	15,4744	15,3561	4,0978	15,0220	14,8519	3,1972
IL-22	94,7669	95,4454	23,8941	88,8639	90,4316	15,7027
IL-23	42,4421	70,4735	79,0737	32,7100	62,5360	70,2685
IL-25	12,4906	12,7253	2,4403	12,4906	11,9548	2,3814
IL-31	344,0335	295,6018	217,0729	294,2489	230,7415	200,8768
sCD40L	88,2887	175,2647	278,9027	92,7544	147,5596	183,6186
TNF-alpha	0,0000	5,1769	11,8366	0,0000	2,2746	3,5073

Figure S1. Detailed comparison of serum cytokine concentrations between the allergy group (1) and

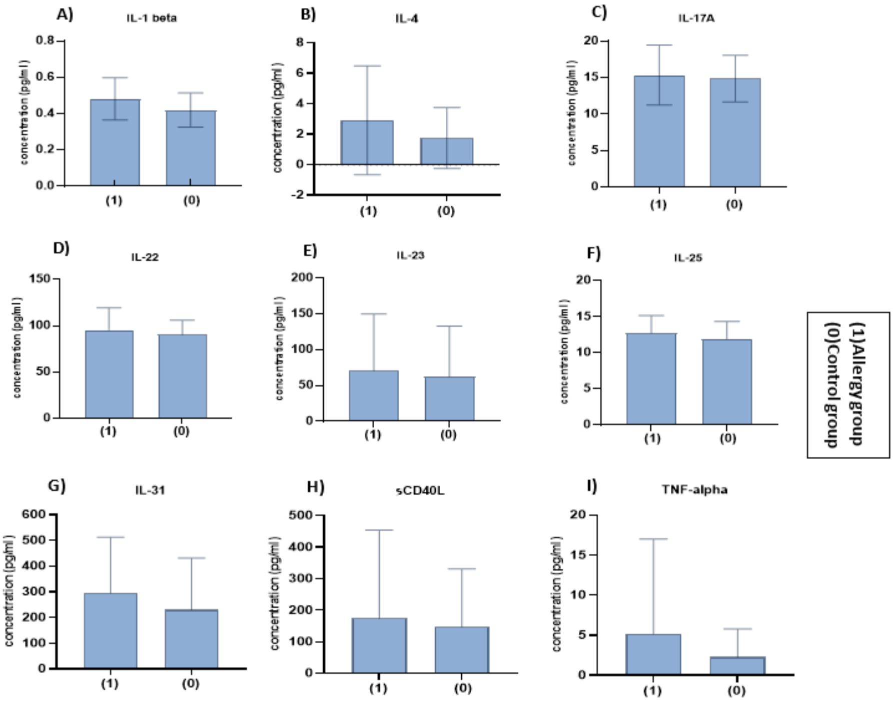

the control group (0).

Table S2. Concentration values of nine cytokines in the IgE-mediated allergy group (1), the delayed-type allergy group (2), and the control group (3). The numerical variables are given in pg/ml.

Cytokine	IGE-MEDIATED ALLERGY GROUP (1)			DELAYED-TYPE ALLERGY GROUP (2)			CONTROL GROUP (3)		
	Median	Mean	SD	Median	Mean	SD	Median	Mean	SD
IL-1beta	0,5136	0,4811	0,1160	0,4361	0,4239	0,1007	0,4168	0,4151	0,0923
IL-4	2,0702	2,9160	3,5823	2,0702	1,5396	1,4369	1,4210	1,8751	2,2940
IL-17A	15,4744	15,3561	4,0978	14,7437	14,4977	3,1952	15,1139	15,0644	3,2450
IL-22	94,7669	95,4454	23,8941	85,7783	88,9757	19,1280	91,8569	91,3052	13,6064
IL-23	42,4421	70,4735	79,0737	16,3665	44,7748	57,6835	61,7873	73,1927	75,9425
IL-25	12,4906	12,7253	2,4403	12,4906	11,7720	2,3634	12,4906	12,0644	2,4339
IL-31	344,0335	295,6018	217,0729	280,8525	213,5733	213,2134	294,2489	241,0424	196,8707
sCD40L	88,2887	175,2647	278,9027	91,2690	147,6732	209,8282	94,2398	147,4914	170,6026
TNF-alpha	0,0000	5,1769	11,8366	0,0000	2,1080	3,8241	0,0000	2,3746	3,3810

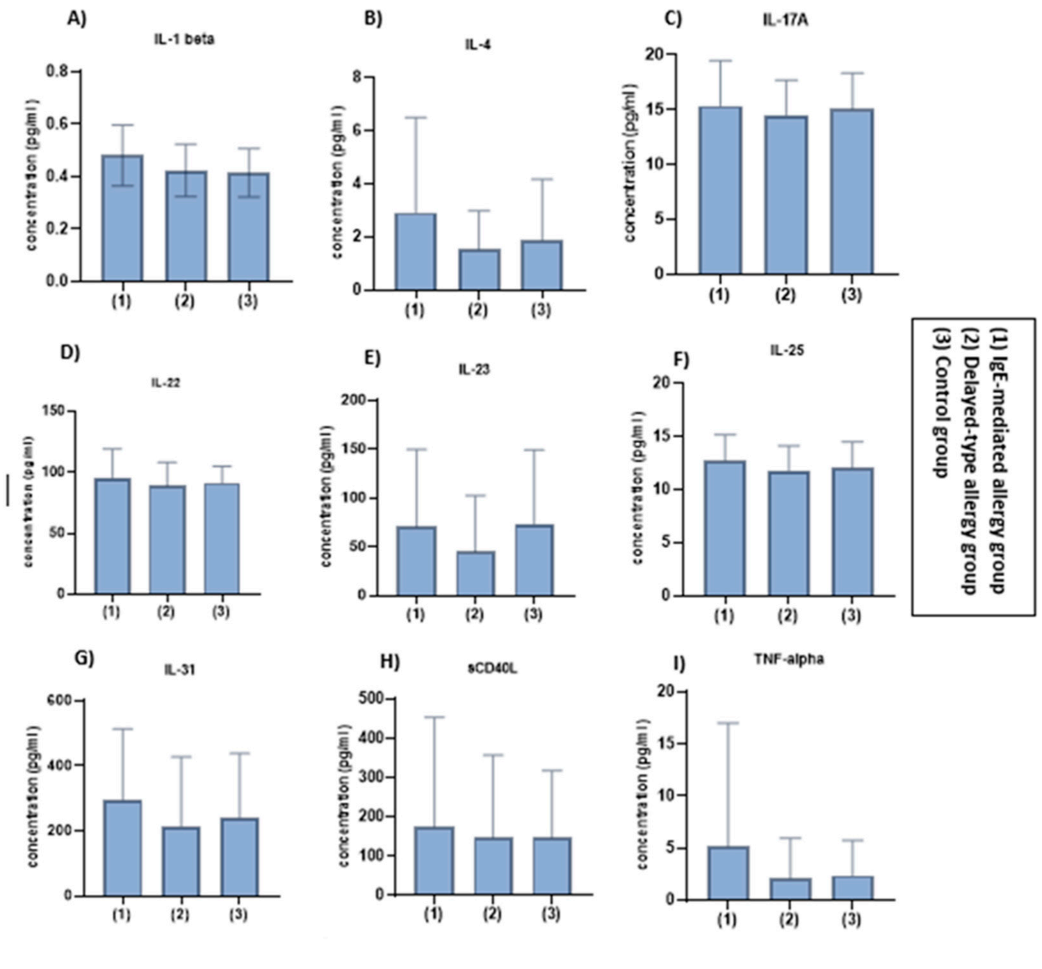

Figure S2. Detailed comparison of serum cytokine concentrations between the three studied groups: the IgE-mediated allergy group (1), the delayed-type allergy group (2), and the control group (3).
